# Supplementary material for: Are carbon emissions trading and green financial instruments synergistic? -Comprehensive quantitative research based on content analysis
Source: PLoS One. 2024 Mar 7;19(3):e0298601. doi: 10.1371/journal.pone.0298601 (PMC10919601; doi:10.1371/journal.pone.0298601)
Supplement: S1 Data — (ZIP) [file pone.0298601.s001.zip › Data/地级市Z_F工作报告.zip!/─┌├╔╣┼/╬┌└╝▓∞▓╝╩╨/└┤╫╘╣1⁄2╓┌║┼í╛┬φ┐╦╩2╛▌═°í┐_2016.docx]

各位代表：

现在，我代表市人民政府向大会报告工作，请连同《乌兰察布市国民经济和社会发展第十三个五年规划纲要(草案)》一并审议，并请市政协委员和列席会议的同志们提出意见。

一、“十二五”时期和2015年工作回顾

过去五年，是我市发展进程中具有里程碑意义的五年。在自治区党委、政府和市委的正确领导下，市政府团结带领全市各族干部群众，全面贯彻党的十八大和十八届三中、四中、五中全会精神，全面贯彻习近平总书记系列重要讲话特别是考察内蒙古重要讲话精神，协调推进“四个全面”战略布局，坚持破除“三论”、树立“三观”，围绕“五个发展定位”，以“三篇文章”“五城联创”和“十个全覆盖”为主抓手，在解放思想中开拓创新，在攻坚克难中砥砺奋进，实现了经济大发展、城乡大改观、民生大改善、形象大提升。

(一)综合经济实力跃上新台阶。始终坚持发展第一要务，主动适应经济发展新常态，狠抓重点工作、重点工程和重大举措，千方百计做大经济总量，提高发展质量，实现了“十二五”圆满收官。2015年，预计完成地区生产总值921亿元，是“十一五”末的1.6倍，年均增长9.2%；固定资产投资658.5亿元，是“十一五”末的2.4倍，年均增长 19.1%；一般公共预算收入55.1亿元，是“十一五”末的3.2倍，年均增长 26.1%；社会消费品零售总额290.7亿元，是“十一五”末的1.8倍，年均增长 13%。主要经济指标增速连续五年位居全区前列，经济发展步入总量扩张、增速加快、质量效益同步提升的新阶段。

(二)产业转型升级迈出新步伐。围绕“六大基地”“五大支柱产业”“四大新兴服务业”，制定完善产业发展规划和扶持政策，在稳定增长中调结构、增效益，在创新驱动中抓转型、促升级，实现了新常态下的新发展。

农牧业规模与效益同步增长。坚持走特色路、打绿色牌，形成了薯、菜、乳、肉等优势特色产业，绿色农畜产品基地建设成效显著。以“高效设施化”引领农牧业现代化。高效节水灌溉面积达到255万亩，设施蔬菜17.3万亩，均比“十一五”末增长90%。建成250公里马铃薯产业带，优质种薯生产体系不断完善。农牧业综合机械化水平达到78%，农牧业现代化进程全面加速。以“生产标准化”保障农畜产品优质化。创建马铃薯、蔬菜标准化生产基地110万亩，畜禽标准化养殖场813个。良种覆盖率达到97%。“三品一标”产品数量实现翻番，达到228个。国家农业科技园区获批建设。“乌兰察布马铃薯”成为全区唯一入选“中国品牌价值评价信息榜”的区域品牌。以“种养规模化”推进农牧业产业化。家庭农牧场和种养大户发展到4681户，国家级合作示范社19家、自治区级34家，种养业规模化经营比例分别达到24%、57%。蓝威斯顿、雏鹰等一批龙头企业相继落地，农畜产品加工转化率由35%提高到53%。农牧民来自产业化经营的收入达到40%。

工业经济总量与质量同步提升。坚持存量提质、增量做优，推动产业延伸、升级、多元，六大主导产业成为引领全市经济增长的支柱产业，氟化工、PVA、石墨碳素等产业处于全区领先水平。千万千瓦级清洁能源基地全面建成，电力总装机达到1255万千瓦，占全区的12%。冶金、化工等传统产业实现延伸升级，石墨新材料、云计算等新兴产业发展态势良好。伊东、蒙维、华为等一批重点项目相继落地投产，销售收入超亿元的企业达到236户，营业收入超百亿元的园区达到3个。工业固定资产投资年均增长17.5%，累计完成1724亿元，是“十一五”时期的3倍。规模以上工业增加值年均增长12.8%。

服务业发展速度与比重同步提高。区域性商贸物流中心建设强力推进，集宁现代物流园区被评为全国优秀物流园区，综合物流产业园列为国家级煤炭物流服务业标准化试点项目，我市成为全区现代物流业改革创新试点地区。维多利、红星美凯龙、康泰惠民园等一批商业综合体建成运营。区域性草原文化旅游度假基地建设步伐加快。“一心两翼三大景区”发展格局全面确立，旅游接待人数和总收入均比“十一五”时期增长3倍。凉城旅游业列为自治区70周年献礼工程，我市被命名为“中国草原避暑之都”。“四大新兴服务业”破题起步。金融机构存贷款余额比“十一五”末翻了一番。服务业对经济增长的贡献率达到36%。

(三)城乡建设实现新跨越。围绕打造区域性中心城市，“五城联创”全面启动，宜居宜业宜游成为城市新名片，人民群众的归属感、获得感、自豪感极大增强。城镇化率由42.3%提高到47%。城市建设突飞猛进。坚持高起点规划、高标准建设、高水平管理，累计完成城建投资1000亿元，是“十一五”时期的3.4倍。各旗县市城镇持续扩容提质，城镇体系更加健全，承载能力显著增强。中心城区形成了“一市两区三组团”框架，确立了城市百年发展架构，建成区面积扩大到68平方公里，城市道路、集中供热、垃圾污水处理、绿化等指标均达到或超过自治区平均水平。通过单位包联、全民共建，形成强大创城合力，中心城区553个住宅小区实现了硬化、绿化、亮化、净化、美化全覆盖，城市形象和宜居指数大幅提升。“三山两河”成为城市标志性景观，获得自治区“人居环境范例奖”。我市被评为全国可再生能源建筑应用示范城市。成功创建“国家园林城市”，被誉为“建在玄武岩上的美丽园林城市”。农村牧区“十个全覆盖”工程扎实推进。着眼统筹城乡发展，补齐农村牧区基础设施和公共服务短板，累计投资70亿元，覆盖78%的行政村、近50%的自然村，90万农牧民直接受益，农村牧区生产生活条件实现历史性飞跃。广大妇女充分发挥“半边天”的作用，积极参与“十个全覆盖”工程建设。19个村镇荣获“国家历史文化名镇”“国家传统村落”和“国家重点示范镇”等称号。基础设施建设全面加强。累计完成道路交通投资580多亿元，是“十一五”时期的4倍。重载运煤专线、汽车客运总站等一批重点项目建成投运，高等级公路里程居全区第一，嘎查村沥青水泥路通畅率达到86%。呼张客专建设顺利推进，集大高铁列入国家中长期铁路网规划，集宁机场春节前通航，现代化立体交通体系更加完善。电网、水利、信息等基础设施保障能力明显增强。生态建设和保护力度空前。累计投入189亿元，完成国家和地方重点生态工程827万亩。沿公路干线两侧50米，建成2566公里绿色生态长廊。每个旗县建成一个万亩义务植树基地和一个万亩生态公园。中心城区累计栽植树木3000多万株(丛)，是“十一五”时期的5.7倍。禁牧休牧效果明显，生态自我修复能力显著增强。森林覆盖率由21.9%提高到23.1%。

(四)改革开放取得新突破。以经济体制改革为牵引，各领域改革持续深化，形成了一批有特色、可复制、可推广的范例。简政放权成效明显。文化、食药、工商、质监等管理体制和机构改革全面完成。前旗、丰镇被确定为全区扩权强县试点。市县两级行政权力清单全部向社会公开，市本级行政权力事项减少35%，行政审批平均时限由18.5个工作日缩短到5.5个工作日。重点领域改革稳步推进。“三证合一、一照一码”工商登记制度改革全面实施，建成全区首个市场诚信平台。土地、草牧场确权试点和基本草原划定工作取得阶段性成果。土地流转面积达到34%，居全区前列。四子王旗被确定为全区唯一的县级国家主体功能区试点。政府和部门预决算、“三公经费”预决算全部向社会公开。2家农信社转制为农商行。“三篇文章”实现精彩开局。乌大张合作区建设纳入国家京津冀协同发展规划，合作区规划和实施方案正式出台，各领域合作深入推进。京蒙对口帮扶合作扎实开展，中关村科技园、乌兰察布大兴产业园等合作共建园区，华为云计算等一批重点项目相继落地实施。集宁海关正式开关，向北开放格局更加完善。

(五)民生保障水平得到新提高。坚持保基本、兜底线、建机制，织密织牢民生保障网，财政用于民生的支出达到86.7%。城乡居民收入实现翻番，年均分别增长11.8%和14%。扶贫攻坚成效显著。“三到村三到户”精准扶贫、金融扶贫和生态移民扶贫工作扎实开展。累计投入财政专项扶贫资金20.3亿元，是“十一五”时期的6.5倍，撬动金融扶贫资金27亿元，有力地支持了贫困地区发展。贫困农牧民由2011年底的55.8万人减少到15万人。就业创业扎实推进。积极引导和扶持大众创业、万众创新，建成就业创业孵化园24个，“乌兰美”成为自治区级劳务品牌。转移农牧民就业72万人，新增城镇就业8.2万人，城镇登记失业率控制在4%以内。社会保障水平全面提升。城乡居民养老保险制度实现全覆盖，机关事业单位养老制度改革稳步推进，企业退休人员养老金逐年提高。新农合政策范围内住院报销比例达到75%。在全区率先实施互联互通惠民工程，率先启动城镇医保监控系统建设，率先将所有恶性肿瘤纳入大病报销范围。实现呼包异地参保与就医即时结算。保障性住房开工16.8万套，基本建成12.7万套，建设规模居全区首位。农村牧区危房改造完成14.8万户，幸福院互助养老政策被评为“中国十大创新福利政策”。民政保障各类困难人员63万人，补助标准逐年提高。“三个一”民生工程深入实施，惠及31.2万户农牧民、8960名大学新生和320户零就业家庭。我市跻身“中国十佳食品安全城市”“全国五星级慈善城市”和“全国最具幸福感城市”。

(六)社会事业呈现新局面。教育事业快速发展。坚持超前、适度负债、标准化办教育，财政用于教育支出200多亿元，新建改建中小学、幼儿园107所，新增校舍面积50多万平方米。教育“三项改革”成效显著，跻身全国十大课改区域样板示范区。民族学校蒙语授课学生率先实现免费教育全覆盖。现代职业教育体系建设和高等教育转型发展迈出新步伐。科技创新作用明显。围绕主导特色产业，建成“一园两院三站八中心”等一批科技创新平台载体，累计实施国家和自治区科技项目153项，科技支撑能力进一步增强。医疗卫生体系更加健全。公立医院改革进展顺利，旗县医院全部实行药品零差率销售，5所县级医院创建二级甲等医院。各级各类医疗卫生机构达到1981个，市中心医院门诊楼、蒙中医院等重点工程建成投用。基本公共卫生服务和食药监管工作跃居全区前列，出生人口素质不断提高。文体事业繁荣发展。建成基层文化活动场所1654个，《忠勇察哈尔》等一批原创优秀文艺作品在全国、全区获奖。国际马拉松、亚洲女子拳击锦标赛等重大赛事成功举办，46所中小学成为国家级校园足球特色学校。民族宗教工作和国防建设成效显著。平安乌兰察布建设扎实推进，安全生产形势保持稳定，信访工作不断加强，社会治安秩序持续向好，人民群众的安全感、满意度大幅提升。统计、粮食、气象、地震、人防、红十字、妇女儿童、残疾人等事业得到长足发展。

(七)政府自身建设取得新成效。认真落实中央“八项规定”和国务院“约法三章”，严守党的政治纪律和政治规矩。深入开展党的群众路线教育实践活动和“三严三实”专题教育，机关干部作风明显好转，行政效能和政府执行力全面提升。扎实推进依法行政和科学民主决策，自觉接受市人大的法律监督和政协的民主监督，人大代表议案、建议和政协委员提案办结率达到100%。严格落实党风廉政建设主体责任，加大政务公开和行政监察、审计监督力度，政府系统廉政建设扎实推进。

各位代表!过去五年，我们迎难而上、负重前行，脚踏实地、真抓实干，谱写了乌兰察布发展史上光辉灿烂的壮丽篇章。五年的不懈奋斗，我们实现了经济社会跨越发展，创新完善了发展思路、厚植了发展优势，干部群众的精神状态、思想观念、文明素质极大提升，为“十三五”奠定了坚实基础。这些成绩来之不易，是自治区党委、政府和市委科学决策、正确领导的结果，是全市各族人民守望相助、团结奋斗的结果。在此，我代表市人民政府，向所有关心、支持和参与乌兰察布改革发展的同志们、朋友们，表示衷心感谢并致以崇高敬意!

回顾五年来的工作，我们也清醒地看到，前进的道路上还有不少困难和问题。主要是：经济总量不大，产业层次较低，发展不平衡、不协调问题仍然突出；基础设施支撑保障能力不足，生态环境仍比较脆弱；部分低收入群众生活还比较困难，公共产品供给能力和公共服务水平与人民群众的需求仍有较大差距；政府自身建设和依法行政能力有待进一步加强。对此，我们必须高度重视，采取有力措施认真加以解决。

二、“十三五”时期经济社会发展的主要目标和任务

“十三五”是全面建成小康社会的决胜阶段，也是我市实现赶超跨越的战略机遇期和黄金发展期。我们要树立强烈的使命意识、发展意识和机遇意识，凝心聚力，乘势而上，顺应人民群众过上美好生活的新期待，推动经济社会发展再上新台阶。

根据市委《关于制定国民经济和社会发展第十三个五年规划的建议》，全市经济社会发展的总体要求是：深入贯彻习近平总书记系列重要讲话和考察内蒙古重要讲话精神，认真落实中央、自治区和市委的决策部署，按照“四个全面”战略布局，牢固树立创新、协调、绿色、开放、共享发展理念，主动适应经济发展新常态，坚持以改革开放为动力、以转变发展方式为主线，坚持发展第一要务，坚持稳增长、调结构、惠民生、防风险的总体思路，深入实施“十个全覆盖”“三篇文章”和“五城联创”三大战略，破解发展难题，提高投资有效性，加快培育新的发展动力，改造提升传统比较优势，着力打造“六大基地”、做强“五大支柱产业”、培育“四大新兴服务业”，推动大众创业、万众创新，全力打赢脱贫攻坚战，全面提升民生保障水平，保持经济社会持续健康发展，确保如期全面建成小康社会，把祖国北疆风景线打造得更加亮丽。

主要奋斗目标是：

经济增长速度高于全区平均水平，地区生产总值年均增长8%，一般公共预算收入年均增长8%，固定资产投资年均增长13%，社会消费品零售总额年均增长10%，城乡居民人均可支配收入年均分别增长9%和10%，达到或接近全国平均水平。节能减排指标控制在自治区确定的目标之内。

主要任务和措施是：

(一)坚持创新发展，着力提高经济发展的质量和效益。把创新摆在发展全局的核心位置，推动科技、产业、品牌等全面创新，形成以创新为引领的经济体系和发展模式。科技创新能力明显增强，贡献率达到53%。发挥投资、消费、出口的拉动作用，深化供给侧改革，优化要素配置，培育发展新动力。进一步理顺政府和市场的关系，增强各类市场主体活力，推进重点领域和关键环节改革取得决定性成果，不断拓展发展新空间，构建产业新体系。大力发展互联网经济，推动三次产业发展与信息化深度融合。加快推进农牧业现代化进程。以“五个农牧业”为方向，着力构建粮经饲统筹、农林牧结合、种养加一体、一二三产融合的发展机制。到2020年，马铃薯种薯面积稳定在80万亩，蔬菜100万亩，以燕麦为主的杂粮杂豆达到300万亩，生猪、肉羊形成年出栏670万口、1000万只的生产能力，农畜产品加工转化率达到68%。着力推动工业经济转型升级。坚持新型工业化发展方向，加快推进传统产业新型化、新兴产业规模化、支柱产业多元化，打造清洁能源、高端化工、冶金建材、农畜产品加工、装备制造、战略性新兴产业等优势产业集群。到2020年，电力装机达到2600万千瓦，天然气日处理能力1500万立方米，化工和冶金综合产能分别达到949万吨和1100万吨，规上工业增加值年均增长10%。不断提高服务业发展水平。紧紧围绕首都这个核心市场，推动文化、体育、医疗、养老四大新兴服务业快速发展。加快“三个交易中心”建设，建成临空产业园、保税仓库、保税物流园区，形成国际物流与国内物流有机衔接，大宗商品与散货物流多头兼顾，多式联运、物联网广泛应用的现代物流产业体系，打造国家级现代物流基地和区域性商贸物流中心。抓住北京—张家口冬奥会的契机，借助长城金三角合作机制，着力打响“中国草原避暑之都”旅游品牌，全面提升旅游业综合实力和竞争力，建成京津冀晋旅游休闲度假基地。旅游人数和总收入分别达到2000万人、300亿元。

(二)坚持协调发展，切实增强可持续发展能力。坚持区域协同、城乡一体、物质文明和精神文明并重，完善促进协调发展的政策措施和体制机制，不断增强发展的整体性。统筹推进城乡发展。全力构建“一核两轴多节点”的城市格局，着力优化村镇布局，城镇化率达到55%以上。全面推进“五城联创”，到2017年完成创城并争创“国家生态园林城市”，到2020年创城成果得到进一步巩固和发展。加强市政公用设施和公共服务设施建设，推进城际轻轨、城市水系、地下综合管廊和“海绵城市”建设，提高城市综合承载能力。深入实施农村牧区“三三制”发展战略，有序推进农牧业转移人口市民化。巩固提升“十个全覆盖”工程，健全农村牧区基础设施和公共服务投入管理长效机制。着力完善基础设施。构建现代交通运输体系，旗县市区通一级公路，苏木乡镇通三级公路，中心城区建成环城高速，县城全部建成环城快速路，高等级公路达到4500公里。推进京呼、集大高铁建设，争取北京——莫斯科等国际铁路经停乌兰察布，构建环绕“金三角”、联通呼包鄂、对接京津冀、连接俄蒙欧的高铁网络布局。争取集宁机场升格为国际机场，凉城等5个旗县通用机场建成使用。加快综合交通枢纽和高铁站区商圈建设，创建全国综合交通运输服务示范城市。加快电力外送通道建设，开展区域微型电网试点，构建智能电网体系。实施“气化乌兰察布”工程，实现旗县城镇天然气全覆盖。加快“引黄济岱”等重大水利工程建设，增强发展保障能力。(三)坚持绿色发展，着力改善生态环境。坚持保护优先、自然恢复为主，深入实施山水林田湖生态保护和修复工程。严格落实基本草原保护制度，持续推进禁牧、休牧、轮牧和草畜平衡，用最严厉的执法保护生态建设成果，主要生态系统步入良性循环。以“两带三廊六大区域”为重点，推进林业重点工程建设，森林覆盖率达到25.1%。落实主体功能区规划，严守生态红线，建立完善生态空间治理体系。实行最严格的环境保护制度，坚决打好大气、水、土壤污染防治三大战役。推动低碳循环发展，有效控制能源和土地、水资源消耗，大幅减少主要污染物排放总量，空气优良天数年均达到300天以上，让青山常在、绿水长流、蓝天永驻。

(四)坚持开放发展，持续提高发展的活力和动力。抓住国家京津冀协同发展、“一带一路”和“中俄蒙经济走廊”建设的重大历史机遇，创新完善“三篇文章”战略布局。蒙晋冀(乌大张)长城金三角合作区规划全面落实，产业、服务和基础设施协作取得新突破，建成京津冀协同发展的功能协作区和国家区域合作的示范区。坚持围绕首都、依托首都、保障首都、服务首都、得益于首都，深度融入京津冀协同发展，建成承接首都经济圈产业转移的示范区、产业升级的助力区、产业发展的拓展区。宽领域对接俄蒙欧，加快基础设施和口岸建设，善丹呼日勒口岸开通并逐步向自贸区过渡，打造自治区重要的进口资源深加工基地，建成一批面向俄蒙欧的经济技术合作区、进出口商品加工区。

(五)坚持共享发展，不断增进人民群众福祉。按照人人参与、人人尽力、人人享有的要求，坚守底线、突出重点、完善制度、引导预期，注重机会公平，实现各族人民共同迈入全面小康社会。持续增加公共服务供给，健全就业、教育、医疗、文化、科技、住房和社会保障等公共服务体系，积极推进大众创业、万众创新，不断提高居民收入水平。义务教育实现均衡发展，学前三年入园率达到95%，高中阶段毛入学率90%，自治区示范性普通高中达到8所，教育教学质量进入全区前列，普职比达到5∶5。每个家庭拥有一名合格的签约医生，每个居民拥有一份电子健康档案。各旗县市全部建成标准化文化体育场馆。办好自治区“十四运”，大力发展足球事业。开创民族团结进步新局面。创新社会治理机制，促进社会和谐稳定，建设“平安乌兰察布”。

贫困不除，奋斗不止。今后五年，我们要按照“六个精准”要求，实施好“五个一批”工程，坚决打赢脱贫攻坚战，确保2020年贫困人口全部脱贫，贫困旗县全部摘帽，决不让一户群众在小康路上掉队。

各位代表!展望“十三五”，我们站在更高的历史起点上开启新的伟大征程，前景十分广阔，目标催人奋进。我们坚信，经过全市各族人民的共同努力，五年的宏伟蓝图一定能如期实现，乌兰察布的明天一定会更加美好!

三、2016年的主要工作任务

2016年是“十三五”规划的开局之年，是推进结构性改革的攻坚之年，也是迎接自治区成立70周年的重要一年。按照全区经济工作会议精神和市委三届六次全委(扩大)会议的总体部署，经济社会发展的主要目标是：地区生产总值增长8%，固定资产投资增长13%，一般公共预算收入增长8%，社会消费品零售总额增长9.5%，城乡居民收入分别增长9%和10%。

重点抓好十个方面的工作:

(一)深入推进改革开放

以供给侧结构性改革为重点，加大改革攻坚力度。准确把握中央和自治区供给侧结构性改革的政策思路，坚决贯彻“宏观政策要稳、产业政策要准、微观政策要活、改革政策要实、社会政策要托底”五大政策支柱和“去产能、去库存、去杠杆、降成本、补短板”五大任务，提高供给体系质量和效益，增强持续增长动力。开展降低实体经济企业成本行动。落实好降低企业税费负担、社会保险费、财务成本、电力价格、物流成本等政策措施，打好“组合拳”，不断增强企业的盈利能力和发展后劲。进一步简政放权。重点推进“三单一网”改革，完善权力清单，制定负面清单，市本级责任清单6月底前全部公开，加快网上审批系统建设，实现所有事项全流程、并联式网上公开审批。市县乡村四级政务服务体系实现全覆盖。健全现代市场体系。进一步放宽市场准入，完善招商引资和承接首都产业转移一揽子政策，形成一整套极具吸引力的政策、服务、保障体系。加快企业信用信息公示系统和诚信平台建设。深化财税体制改革。完善政府预算体系，加强预算执行管理。规范地方政府债务管理。推进政府购买公共服务工作。加快投融资体制改革。设立基础设施和产业发展基金，大力推广PPP合作模式，改进政府投资方式。健全多层次资本市场体系，推动企业上市，提高直接融资比重。加快国有担保体系建设，扩大中小企业风险池基金规模，实现县域全覆盖。兴和农信社筹建农商银行。深化农村牧区综合改革。完成草牧场确权登记，土地确权工作全面推开。大力发展适度规模经营，土地和草牧场流转面积分别达到410万亩和480万亩。

以“三篇文章”为统领，全方位扩大开放。积极推进蒙晋冀(乌大张)长城金三角合作区建设。推动建立合作区省级协调机制，争取合作区规划通过国家审批。联合申报国家生态安全屏障示范区、国家集中连片特困地区扶贫攻坚示范区。推动集大高铁、合作区天然气等重大项目建设。促进乌兰察布综合物流园等合作园区建设，实现产业深度合作。主动融入京津冀协同发展。以教育、医疗、扶贫为重点，推动京蒙对口帮扶合作精准化。将2016年确定为“承接首都产业转移攻坚年”，理清承接产业目录，明确“一对一”承接领域，落实责任部门和牵头领导，强化实绩考核，干部在京挂职期间每人要至少引回一个项目。大力发展“飞地经济”，加快中关村科技园、察右前旗与北京大兴区共建产业园等平台建设，落实好总投资255亿元的40个重点合作项目。加快实施向北开放战略。主动融入“一带一路”建设，大力推进善丹呼日勒口岸前期工作，力争与蒙古国东戈壁省签订边民通道开放协议，开拓中蒙通关贸易。

(二)着力培育新的经济增长点

准确把握新常态下国家、自治区投资政策导向，围绕补短板、强基础、调结构、增后劲，着力优化投资结构，不断增加有效投资，形成政府投资、企业投资、市场融资、招商引资的聚合效应。实施亿元以上重点项目279个，总投资2214亿元，当年计划完成投资523亿元。围绕提升产业层次，实施雏鹰生猪等农牧业重点项目19个，完成投资27亿元；华为云计算等工业重点项目101个，完成投资246亿元；集宁古城等商贸物流重点项目32个，完成投资42亿元。围绕改善发展环境和条件，实施重点项目88个。交通建设重点推进省道22线白永高速、机场快速路等27个项目，中心城区绕城高速和四子王、卓资、商都、化德绕城快速路建成通车。基本实现嘎查村通沥青水泥路。加快推进京呼高铁建设，积极争取土牧尔台—善丹呼日勒等铁路开工建设，新增一批城际铁路项目。争取集宁机场扩建工程年内完工，为开通大型货运包机创造条件。开工建设凉城、四子王、化德通用机场。做好乌兰察布——华北、四子王旗——武汉、凉城——北京电力外送通道项目前期工作。实施36项大中型电网工程，新增变电容量367万千伏安。隆胜、七家营、红格尔3座中型水库建成蓄水，启动岱海补水工程，争取丰镇市头道沟等8座小型水库开工。加强通讯基础设施建设，实现移动通讯全域无盲区。围绕保障和改善民生，实施民生社会重点项目39个，完成投资81亿元，突出抓好移民搬迁、保障性住房和“十个全覆盖”工程。适应消费趋势变化，在释放汽车、住房等刚性消费需求的同时，努力发展新型消费业态，引导消费朝着智能、绿色、健康、安全方向转变，推动消费潜力不断释放。

(三)加快转变农牧业发展方式

强化新科技的引领作用、新主体的核心作用和新方式的促进作用，加快转方式、调结构、强基础步伐，走产出高效、产品安全、资源节约、环境友好的现化农牧业发展路子，突出抓好“五个农牧业”，建设绿色农畜产品生产加工输出基地。

大力发展特色农牧业。围绕马铃薯、冷凉蔬菜、以燕麦为主的杂粮杂豆、生猪、肉羊等五大主导产业，推进规模化布局、标准化生产、集约化经营。优质马铃薯面积稳定在400万亩，冷凉蔬菜达到60万亩，燕麦扩大到100万亩，生猪和肉羊年出栏分别达到200万口和800万只。

加快发展设施农牧业。围绕国家农业科技园区，建设前旗超时令蔬菜基地、凉城生态观光农业带、丰镇错季水果产业园、商都有机瓜果产业园等一批设施农业示范区，创建蔬菜标准园10个。以温室和大棚为主的设施农业达到25万亩。大力发展舍饲畜牧业，生猪、肉羊规模化养殖比例分别达到70%和35%。

做大做优种子农牧业。着力推进马铃薯产业转型升级，引进蓝威斯顿种薯生产管理模式，建设育繁推一体化种薯企业，供种能力达到150万吨。扶持赛诺、鹏程等畜种企业加快新品种引进、选育和示范推广工作。实行最严格的市场准入和行业监管，加快主导产业良种化扩张，建设辐射全国的优质种薯种畜生产基地。

提升发展绿色农牧业。实施土壤健康工程，减少化肥农药不合理使用，严格执行土地倒茬轮作。推行绿色标准化生产，实现重点区域和国家农业科技园区全覆盖。加大“三品一标”认证力度，新增认证面积8万亩，认证无公害、绿色、有机农畜产品20个，“四子王戈壁羊”“乌兰察布燕麦”“商都西芹”通过国家地理标志证明商标认证，民丰、赛诺打造国家驰名商标。健全从农田到餐桌的农产品质量监管体系，30家企业纳入自治区可追溯信息平台，推动名优特产品出区进京免检。鬼吹灯小说

延伸发展加工农牧业。坚持用工业化思维谋划农牧业发展，以龙头企业为根本，推动农牧业向价值链高端迈进。每个旗县引进培育2—3家年销售收入5亿元以上的领军企业，农畜产品加工转化率达到56%。加快雏鹰生猪产业化项目建设，打造后旗、中旗、商都三个百万口生猪大县。壮大四子王赛诺、后旗蒙多利两个百万肉羊联合体。推进蓝威斯顿和北京凯达马铃薯、民丰燕麦、田也杂粮杂豆深加工项目，构建全产业链发展格局。鼓励发展农牧民专业合作社和家庭农牧场，创建自治区级示范社30个、国家级示范社15个，紧密型利益联结比例达到40%。

(四)做大做强做优工业经济

围绕优势特色产业，以“扩、提、转、引”为重点，横向延伸抓配套，纵向联合抓升级，推动企业循环式生产、园区循环式链接、产业循环式组合，做好存量调整“加减法”。工业固定资产投资和规模以上工业增加值分别增长13%和9%。

优先发展能源产业。坚持火电先行、风光互补，新增电力装机180万千瓦。火电新增装机70万千瓦，推动集宁京宁、兴和宏大尽快并网发电，加快旗下营电厂建设进度，完善四子王旗白乃庙、岱电三期、京隆二期、内蒙古能建投电厂项目前期工作，力争年内至少落地开工一个电厂。风电新增装机90万千瓦，中节能、大唐国际风电项目加快建设，推动乌兰察布千万千瓦级风电基地列入国家风电发展规划。光伏发电新增装机20万千瓦，与设施农业、新农村建设、扶贫开发相结合，推进前旗香岛和中旗山路等项目建设。天然气日处理能力达到370万立方米，抓好陕京四线和呼张延输气管道乌兰察布段建设，新建6个CNG、LNG天然气项目，延伸发展下游产品，建设“气化乌兰察布”。打造面向首都、服务华北的重要清洁能源基地。

改造提升传统产业。坚持扩规模、延链条、促耦合，推动产业转型升级。氟化工要向氟橡胶等高端产品发展，总产能达到30万吨。推动四子王永和项目尽早启动，丰镇氟化工项目扩大生产。电石化工要加大PVA、PVC、糊状树脂等下游产品开发力度。加快蒙维PVA二期等重点项目建设，电石就地转化率达到54%，综合产能达到430万吨。硅化工要加快开发硅晶材料和有机硅等下游产品。拉动四子王20万吨工业硅和佳辉有机硅项目满负荷生产，有机硅产能达到10万吨。冶金产业要向精密合金冶炼、标准件铸造、型材加工延伸。推进上泰特种合金、旭峰硅锰合金项目投产达效，前旗蒙发、化德天成中低碳锰向特种合金铸造延伸，白乃庙铜业年处理450万吨采选技改扩建项目开工。建材产业要向环保节能方向发展，鼓励利用工业废渣生产水泥和新型墙体保温材料。优化整合石材资源，打造四子王、后旗、中旗、化德采选加一体化示范园。香港森诺年加工50万立方米木材项目投产达效。装备制造业要向高端智能化发展。提高地产风机使用率，风机整机产能达到1840台(套)。加快赣商年产1500万件高新医疗器械项目建设。

培育壮大新兴产业。信息产业要依托华为云计算项目，积极布局“互联网+”新业态，推动网游、手游、基因测序等关联企业向我市集聚，打造察哈尔云计算产业园。加快电子政务云平台建设，建成政务协同办公系统。借助京呼高铁铺设大容量通讯光缆，打造信息高速公路。开工建设中信国安大数据中心和呼叫中心。生物医药产业要加大中蒙医药和医药中间体开发力度，推动福瑞科技园项目建设，京东药业、金莉源药业等项目建成投产。新材料产业要加快兴和新石墨矿勘探开发，推动瑞盛石墨和中元龙港项目建设，加强与国内外科研机构合作，加快研发石墨烯等高附加值产品。

提升园区建设管理水平。突出首位产业，推动错位发展，加大旗县工业园区整合力度，形成“一园多区”发展构架。做大做强化德羊驼绒服装、卓资熏鸡、丰镇月饼、中旗燕麦等特色产业园。理顺管理体制，给予园区更大的自主发展权。加快园区提档升级步伐，力争丰镇氟化园区、后旗建材化工园区升级为自治区级园区。

(五)加快发展现代服务业

坚持“双轮驱动”，推动生产性服务业向专业化和价值链高端延伸，推动生活性服务业向精细化高品质提升。服务业增加值占GDP比重达到37.8%。

做大做强现代物流业。发挥机场、高铁和海关形成的新优势，加快建设物流节点城市，打造自治区融入“中俄蒙经济走廊”和“丝绸之路经济带”的战略核心区。〗推动“三个交易中心”转型升级。煤炭交易中心要大力发展陆港经济，重点推进综合物流产业园与曹妃甸港的战略合作。加快推进“三线一站”、数字化储配煤等项目，建设国家标准煤生产基地。马铃薯交易中心要向农畜产品物流交易中心和电商交易中心发展，推动冷凉蔬菜等特色农畜产品在电子交易平台上线销售。皮革交易中心要以集宁国际皮革城和集宁古城为龙头，以集宁皮件加工园、鑫源通皮件产业孵化园和后旗皮毛工业园为支撑，促进全产业链开发。加快建设临空产业园。科学规划园区功能布局，推动6.5平方公里核心区年内开工并尽快投运。加强与天津港、北京空港、曹妃甸港、二连浩特等陆海空港的合作，设立保税区、保税仓库和海关监管区，引进国内外知名物流企业，积极发展空港经济和国际物流。〗打造高铁站区商务圈。统筹站区、商圈和城市建设，加强高铁、客运中心和机场的对接，推进京呼、集大与北京——乌兰巴托——莫斯科国际高铁的连接。加快高铁站区建设，道路、广场、水系和绿化工程全部完工，确保2017年车站和配楼投入运营，商圈基本形成，打造成立体交通枢纽、中心城区亮点、商务经济高地和面向国际的窗口。

创新发展商贸流通业。大力发展电子商务，加快北方食品电商城等项目建设，集宁国际商贸城等电商产业园启动运行，推动电商产业集聚发展。建成6家牧区商贸服务中心，实现农村牧区便民连锁超市全覆盖。推进恩和世纪广场、金茂国际购物中心、华联优品广场等商贸综合体建设，打造现代服务业集聚区、核心圈。

加快发展旅游业。围绕打响“中国草原避暑之都”品牌，提升旅游产业发展层次，打造“体现草原文化、独具北疆特色的旅游观光休闲度假基地”。提升对接合作水平。借助京蒙帮扶和乌大张区域合作平台，积极参与京张联合办奥活动。开发冬季旅游资源，丰富冬季旅游产品，开辟北京——张家口——乌兰察布——大同无缝对接的奥运旅游黄金线路，把我市打造成为冬奥会的协办地、延伸区。提升景区开发水平。围绕满足游客观光、休闲、度假等复合型需求，引进大型企业集团，推动重点景区高水平、大规模、全要素、整装式开发。加快凉城旅游献礼工程建设，开工建设54个重点项目，完成投资21.7亿元。加强前旗玫瑰营风情小镇、卓资林胡古塞、后旗火山草原等景区建设，推动苏木山、辉腾锡勒等景区升A和格根塔拉草原旅游资源整合。提升管理服务水平。围绕吃住行游购娱，补齐要素短板，完善自驾游服务功能，在重点旗县、线路、景区建设宿营地和加水、加油、维修等配套服务设施，创建“全国自驾旅游示范城市”。加快呼凉等旅游专线公路建设。争取承办自治区第27届那达慕大会。推出一批民族民俗文艺演出剧目，提升旅游文化内涵和游客参与度。加强旅游市场综合执法，营造诚实守信的消费环境。

大力发展“四大新兴服务业”。以吸引首都地区的企业和投资者为重点，通过独资、联合、参股等多种形式开展合作，在培育主体、壮大规模、提升知名度和影响力上取得重大突破。充分挖潜地域文化特征，加快建设集宁路遗址、绥蒙革命纪念园等文化产业项目，着力打造民族文化一条街，扩大和引导文化消费。开展居家养老服务试点工作，拓展政府购买公共服务范围。鼓励企业和社会资本参与养老产业发展，建设面向首都的养老社区，争取列入自治区养老服务业试点城市。积极融入奥运经济，加强体育基础设施建设，打造国家级和区域性的体育训练基地或休闲运动中心，建设距离首都最近的冬季运动基地。与北京共建联办、合作经营一批医疗、康复、保健、疗养机构。探索职高与医院联合办学，加快培养高护、特护、康复等重点急需人才。(六)大力推进新型城镇化进程

认真贯彻中央和全区城市工作会议精神，按照“一个尊重、五个统筹”的要求，抓好规划建设管理三大环节，推动城市发展由外延式扩张向内涵式提升转变，加快建设以人为本、和谐宜居、富有活力的城镇体系。

完善城乡规划体系。坚持城市让生活更美好的理念，尊重城市发展规律，完善城市总体规划，高起点编制全市产业空间布局、城市景观等专项规划。加强城市规划立法，充分发挥规划的引领作用和刚性约束。中心城区启动“海绵城市”专项规划和核心区三维城市数字化研究工作，完成新一轮城市近期建设规划和地下综合管廊、慢行交通系统、停车场、电讯、防灾减灾等专项规划编制工作。各旗县市加强重点集镇和嘎查村规划编制及完善工作，启动特色村落、美丽乡村试点规划编制工作，推进规划管理向乡镇一级延伸。城镇化率达到48.2%。

提高城市建设管理水平。以“五城联创”为抓手，提升城市品位，改善人居环境，努力让人民群众生活得更方便、更舒心、更美好。各旗县市创建自治区级园林、卫生、食品安全城市，有条件的争创国家级城市。完善城市功能。坚持地上地下空间同步开发，新区建设和旧城改造统筹推进，实施市政基础设施建设项目177项，完成投资30亿元。新建改建城区主干道76.5公里，硬化街巷17万平方米。启动友谊大街等5条道路地下综合管廊建设。新建改建水源地5处、供水管网245公里。集中供热和燃气普及率分别达到85%和80%。提升城市品位。把霸王河上下游和南沙河治理作为重点，坚持水、林、路、景、园综合配套，确保当年建成。巩固“国家园林城市”创建成果，突出抓好城市出入口和园林景观打造，新增绿化面积908万平方米，中心城区绿地率达到37.6%。落实好国家、自治区“去库存”政策，加强对房地产市场的引导和调控，回购或回租存量商品房用于保障性住房。完成既有居住建筑节能改造50万平方米以上，同步整治老旧住宅小区，保障性住房全部达到一星级以上绿建标准。争取列入国家住宅产业现代化试点市。加强城市管理。把提供精细化城市管理和良好公共服务作为城市工作的重点，促进城市管理向网格化、智能化、人性化方向发展。确定2016年为“物业管理服务规范年”，制定物业管理条例，实现市域城镇物业管理全覆盖。倡导绿色出行，建设公共自行车换乘系统，实现与公共交通的无缝对接。建设“智慧乌兰察布”，建好城管、交警、应急三大公共服务信息平台，中心城区主要公共服务场所实现免费WIFI全覆盖。提升市民文明素质，调动广大群众参与城市建设管理，迎接“全国文明城市”中期考察验收。

(七)不断加强生态文明建设

加大生态建设和保护力度。实施国家重点生态建设工程101万亩。继续打好“山水绿”三张牌，高标准推进城镇、园区、通道、景区、村屯绿化，完成地方重点区域绿化21万亩，重点流域水保治理27万亩。启动岱海、黄旗海水生态治理工程。严格执行基本草原保护制度，完成草原建设420万亩以上。严格落实禁牧、休牧、轮牧和草畜平衡制度，严禁偷牧行为，一经发现严肃问责。加强地质环境治理，对关闭取缔的矿山、园区全部进行生态恢复。推进四子王旗“国家主体功能区”试点工程，全面改善区域生态环境质量。

强化环境污染防治。围绕创建“国家环保模范城市”，加大环境综合整治和执法力度，切实解决损害群众健康的环境污染问题。加强以燃煤锅炉、机动车尾气为重点的大气污染治理工作，PM2.5浓度下降10%以上。持续推进水污染物减排工作，加强污水管网建设，城镇生活污水处理厂负荷率达到75%以上，提高中水回用率。试行节能降耗季度核算预警制度，全面完成自治区下达目标。

推动资源集约节约利用。大力发展循环经济，抓好农牧业清洁生产、工业循环利用示范项目和基地建设。实行最严格的水资源和耕地保护制度。加大闲置土地清理和废弃居民点、工矿废弃地复垦力度，提高土地供地率和集约化利用水平。

(八)着力提高公共服务均等化水平

优先发展教育事业。继续深化教育“三项改革”，推进教育“管办评”分离改革试点工作。优化学校布局，全面实施“薄改工程”。6个旗县通过义务教育均衡发展达标验收。加快普及学前教育，学前三年入园率达到90%。推动各旗县市区与北京合作办学，实现普通高中优质多样化发展。大力发展现代职业教育，推进民族教育、民办教育、特殊教育协调发展，加快高等教育内涵发展。

大力发展医疗卫生和计生事业。全面推进县级公立医院综合改革，建立科学合理的投入、监管和运行机制，完善医院法人治理结构。不断完善四级医疗卫生服务体系，推行分级诊疗模式，推进医疗资源向基层、向农村牧区倾斜，每个旗县至少选择1个乡镇中心卫生院挂牌成为县级医院分院。保障嘎查村卫生室经费和工资，加强乡村医生规范化、系统化全员培训，提高服务能力。深化京蒙对口帮扶医疗卫生合作，县级综合医院全部与北京三级医院建立“一对一”帮扶协作关系。加强市中心医院领先学科、重点学科和重点实验室建设，创建三级甲等医院。兴和县医院等5所医院创建二级甲等医院。市第二医院等9个项目建成投用，四子王旗蒙中医院等7个项目开工建设。深入开展爱国卫生运动，加强传染病和地方病预防控制。启动“国家卫生城市”申报工作。坚持计划生育基本国策，落实“全面二孩”政策。

统筹发展科技文体等事业。大力实施“重点领域关键技术攻关、实用高新技术成果转化、创新平台载体建设”三大工程，科技贡献率达到45%。深入实施“人才强市”战略，统筹推进各类人才队伍建设。加强公共文化设施建设，市美术馆、博物馆以及丰镇、兴和等旗县的图书馆、文化馆、美术馆建成使用。认真落实全民健身实施条例，重点打造乌兰察布国际马拉松等品牌赛事，精心筹备“十四运”。市网球馆、游泳馆等大型场馆主体完工，每个旗县建成1个标准化足球场。加强国防后备力量建设，促进军民融合式发展。加强民族宗教工作，落实妇女儿童事业发展纲要，统筹做好统计、地震、气象、人防等工作，推动社会全面进步。

大力推进社会管理创新。加强“平安乌兰察布”建设，创新立体化社会治安防控体系，严密防范、严厉打击各类违法犯罪活动。坚持“党政同责、一岗双责、齐抓共管”，毫不松懈地抓好安全生产。加快食品加工园区和农贸市场建设，食品生产、流通、餐饮环节示范创建实现全覆盖。完成“食品药品安全乌兰察布”70%的工作任务，成功创建“国家食品安全城市”。加强和改进信访工作。健全劳动保障监察和争议仲裁处理机制，全面治理拖欠农民工工资问题。加强应急管理，提高突发公共事件应急处置能力。

(九)努力保障和改善民生

深入实施“十个全覆盖”工程。今年是全覆盖的冲刺之年，要按照王君书记“三个不漏”的要求，坚持适地、实用、实效和缺什么补什么的原则，最大限度发挥群众主体作用，全力实施好“十个全覆盖”工程。全面覆盖1652个30户以上自然村。30户以下的1477个自然村向中心村整合，无法整合的重点解决好住房、饮水、出行等问题，延伸文化、教育、卫生等公共服务，实现功能全覆盖、村庄全覆盖、人口全覆盖。充分发动广大妇女积极投身“十个全覆盖”工程建设，参与率和受益率均达到100%。启动实施“美丽乡村”产业基金，吸引社会资金参与农村牧区“十个全覆盖”工程建设。抓好抗旱水源工程和新型水窖建设，切实解决严重缺水嘎查村的饮水安全问题。全面开展农村牧区环境综合整治，选择30个苏木乡镇政府所在地、300个村庄作为试点，建设垃圾、污水收集处理设施，打造干净整洁、环境优美的示范村镇。金庸小说

扎实推进脱贫攻坚工程。落实“六个精准”脱贫措施，实施“五个一批”脱贫工程，必保年内3万人稳定脱贫。发展生产方面。深入实施“一村一品”产业推进行动，引导扶持贫困户因地制宜发展特色种养业。加大金融扶持力度，综合采取财政奖补、风险补偿等方式，撬动金融扶贫贷款14亿元，帮助贫困户解决贷款难、贷款贵的问题，促进贫困人口就地脱贫。易地搬迁方面。生态恶劣地区50岁以下、有劳动能力的贫困人口，集中向小城镇、旅游区和各类园区移民搬迁、安置就业。生态补偿方面。坚持绿色扶贫，推动生态保护补偿政策和生态工程向贫困地区、贫困人口倾斜，积极发展林下经济和后续产业，提高贫困人口参与度和受益水平。教育扶贫方面。加大贫困地区教育投入力度，推进农村牧区义务教育阶段学生营养改善计划。加强乡村教师队伍建设，全面落实连片特困地区乡村教师生活补助政策。落实好中等职业教育扶贫“雨露计划”，实施好贫困家庭大学生入学资助政策，让贫困家庭子女都能平等接受优质教育，阻断贫困代际传递。社会保障兜底方面。将完全或部分丧失劳动能力的贫困人口全部纳入农村牧区低保，其他贫困人口每人每年给予2000元临时救助。借助京蒙对口帮扶医疗技术力量，实施医疗健康精准扶贫工程。力争每年为贫困人口体检一次，建立健康档案。对筛查出的重症患者分类施策，通过专家集中会诊、开设家庭病床、提高新农合报销比例、设立大病保障基金等多种措施，解决贫困人口因病返贫问题。全面建立困难残疾人生活补贴和重度残疾人护理补贴制度。

大力推进大众创业、万众创新。建设康泰惠民园、集宁国际皮革城等重点创业园区，每个旗县至少建设1个容纳500人以上的创客空间园区，市电子商务创业孵化园和集宁师院大学生创业孵化园投入使用，市民族用品加工实训基地建设完工。启动“互联网+就业”服务项目，完成市县乡村劳动保障就业服务平台建设。深入推进就业“六项计划”，统筹做好高校毕业生、农村牧区富余劳动力、城镇困难人员和退役军人就业工作。转移农牧民就业稳定在70万人，新增城镇就业1.6万人，城镇失业登记率控制在3.9%以内。

全面推进百姓安居工程。围绕“住有所居、居有改善”的目标，加强城乡住房保障工作。新建城镇保障性住房2.1万套(户)，基本建成1.8万套(户)。严把补助对象审核审批关，规范信息录入工作，完成农村牧区危房改造4万户。建设互助幸福院1000户，推进规范化管理。

不断提高社会保障水平。实施全民参保计划，建立社保金征缴与财政拨款挂钩机制，依法推进扩面征缴。全面完成机关事业单位养老保险制度改革。健全完善全民医保体系，城镇职工和居民政策范围内报销比例保持在85%和75%以上，新农合市内住院报销比例达到80%。县级新农合信息平台整合为市级统筹平台。深化医保支付方式改革，建立总额控制和住院床日付费制度，单病种付费由16种增加到30种。以互联互通惠民工程为重点，加强基层和外出务工人员服务中心信息化建设，城乡社会保障信息查询、待遇发放等“一卡通”服务实现全覆盖。推动商业保险机构承办大病保险工作。开展城乡低保专项整治“回头看”工作，提高各类民政保障人员补助标准。

(十)全面加强政府自身建设

适应新常态、谋求新发展、完成新任务，对政府自身建设提出了新的更高要求，必须着眼全局、顺势而为。以扎实的举措践行法治。坚持把依法行政贯穿到政府工作的各个领域、各个层面、各个环节，用法治思维和方式履职尽责。严格执行重大事项公示制度、社会听证制度、专家咨询制度和政府法律顾问制度，规范行政决策程序，提高政府公信力和执行力。自觉接受人大的法律监督和政协的民主监督，强化行政监察和审计监督，确保行政权力阳光、公正、规范运行。以优良的作风狠抓落实。自觉践行“三严三实”要求，恪尽职守、敢于担当、主动作为，严肃纠正不作为、乱作为现象，让“忠诚干净担当实干”成为各级干部共同的精神品格、价值追求和行为准则。进一步健全抓落实的体制机制，创新督查方式，保障政令畅通，打通政策落实“最后一公里”。以严明的纪律守住廉洁。坚决贯彻全面从严治党的要求，严格遵守《准则》和《条例》，强化“一岗双责”，落实好党风廉政建设主体责任。严格控制“三公”经费，完成公务用车制度改革。强化重点领域和关键环节的廉政风险防控，坚定不移推进反腐倡廉工作，营造风清气正的政治生态。

同志们，时代赋予重任，拼搏铸就辉煌。新的征程已经开启，新的目标催人奋进，让我们在市委的坚强领导下，团结一致、锐意进取，坚定信心、扎实工作，全力推进全面小康社会建设，以优异的成绩向自治区成立70周年献礼!
